# Does functional strength training program improve ice speed and agility in young elite ice hockey players? Functional strength training’s impact on hockey performance

**DOI:** 10.3389/fphys.2025.1448495

**Published:** 2025-03-12

**Authors:** Anna Bieniec, Małgorzata Grabara

**Affiliations:** ^1^ Department of Health-related Physical Activity and Tourism, Jerzy Kukuczka Academy of Physical Education in Katowice, Katowice, Poland; ^2^ Institute of Sport Science, Jerzy Kukuczka Academy of Physical Education in Katowice, Katowice, Poland

**Keywords:** ice hockey, speed, agility, smart speed system, functional strength training

## Abstract

**Purpose:**

The aim of this study was to investigate the effects of a functional strength training (FST) program on ice speed and agility in young elite male ice hockey players.

**Methods:**

Forty-three ice hockey players, aged 15–18 years participated in the study. The athletes were randomly assigned to either a functional strength training group (FSTG), which completed an additional FST program consisting of two 60-min training sessions per week, or a control group (CG), which participated in other team games and swimming sessions, each lasting 60 min. Ice skating speed was assessed using the professional Smart Speed measurement system in forward and backward skating tests over 5, 15, and 30 m, as well as in an agility test.

**Results:**

The intervention elicited significant performance improvements in ice skating speed and agility in the FSTG compared to the CG. Significant time × group interactions were observed in the 5-m (p = 0.041, *η*_*p*_^2^ = 0.098), 15-m (p = 0.047, *η*_*p*_^2^ = 0.093), and 30-m (p = 0.011, *η*_*p*_^2^ = 0.149) forward skating tests, highlighting differential responses between groups. Post hoc analysis confirmed significant improvements in the FSTG, particularly in the 15-m and 30-m tests, where post-test results were superior to those of the CG. No significant effects were found for the backward skating tests. Regarding agility, a significant main effect of time (p = 0.023, *η*_*p*_^2^ = 0.12) and group (p = 0.001, *η*_*p*_^2^ = 0.226) was detected. In the full speed test, only a group effect (p = 0.026, *η*_*p*_^2^ = 0.116) was observed, with no significant time × group interaction or time effects.

**Conclusion:**

These results underscore the effectiveness of FST in enhancing forward skating speed and agility.

## 1 Introduction

Ice hockey, the fastest team sport in professional athletics, is a high-intensity sport that requires players to skate at high speed, change direction quickly, brake quickly, and engage in frequent body contact. Acceleration, a fundamental skill, plays a crucial role in determining a player’s performance (Montgomery, 2006). Ice hockey requires players to develop muscular strength and power to enhance their speed and agility on the ice (Montgomery, 2006). The fundamental motor skills, which includes coordination, mobility, and stability, forms the foundation for targeted movement patterns necessary for strength and speed training, as well as the sport-specific patterns required during the game (Boyle, 2016).

To assess the impact of the implemented functional strength training (FST) on sport-specific fitness, it was agreed with the head coaches to conduct simple on-ice tests measuring forward speed, backward speed, and agility. These tests were designed to evaluate key performance attributes essential for ice hockey players: optimal acceleration over 5 m and maximum skating speed over 30 m. Additionally, as athletes frequently change direction during play, agility assessment was deemed necessary.

Functional strength training (FST) is a form of motor preparation commonly utilized in contemporary sports practice. It is defined as any type of exercise designed to enhance specific functions, movements, or combinations of movements, while also being individually tailored to address functional deficits in a particular athlete or within the context of a specific sports discipline (Pacheco et al., 2013). The primary aim of FST is to improve sports performance and refine targeted movements by replicating desired actions, rather than isolating specific muscle groups (Akbar et al., 2022; Stenger, 2018). Addressing functional deficits during the developmental period offers significant benefits, potentially contributing to the long-term success of an athlete’s sports career (Boyle, 2016; Xiao et al., 2021).

FST is designed to prepare the musculoskeletal system, as well as the joint and ligamentous structures, to meet the physical demands of training and competition. FST involves a structured and planned sequence of exercises aimed at developing athlete-controlled movement patterns, which serve as a foundation for more complex, integrated movements (Boyle, 2016). Effective FST relies on close monitoring of athletes, including the use of functional assessments such as the FMS and Y-Balance tests to identify deficits and asymmetries. Ice hockey, characterized by its asymmetric movement patterns and a strong reliance on flexion, presents unique challenges. Not all strength exercises with additional loads, commonly employed in traditional strength training, are appropriate for hockey players, as these may result in incomplete execution or compensatory movements. Furthermore, due to the high demands placed on specific muscle groups during on-ice activities, additional off-ice strength training targeting these same groups may exacerbate overuse and is therefore unnecessary (Bashir et al., 2022; Pacheco et al., 2013).

Improving athletic performance requires the continuous development of speed and jumping capabilities. An increasing number of studies focus on the application of FST not only for injury prevention but also to optimize motor skills across various sports disciplines (Gambetta, 2007; Xiao et al., 2021). Most studies report positive effects of FST on sprinting, jumping, and functional movement patterns, as assessed using the Functional Movement Screen (FMS™) (Baron et al., 2020; Bashir et al., 2022; Bhardwaj and Kathayat, 2021; Vagner et al., 2022; Yildiz et al., 2019). For instance, the study involving young hockey players demonstrated significant improvements in the overall FMS™ score, as along with enhanced performance on the FMS asymmetry tests, after completing a 12-week FST program (Bieniec and Grabara, 2024). Similarly, improvements in physical fitness have been observed in basketball players following the implementation of integrated FST (Gambetta, 2007), as well as in athletes from other sports (Fort-Vanmeerhaeghe et al., 2016). However, to the best of our knowledge, studies examining the effects of FST on specific fitness components in ice hockey, particularly on-ice performance, remain limited (Bashir et al., 2022). Keiner et al. (2024) investigated the influence of maximum strength and power performance on linear on-ice skating performance both during testing and game play, conducting a cross-sectional study with 24 highly trained male youth ice hockey players. Similarly, Dæhlin et al. (2017) compared the effects of combined plyometric and strength training on skating sprint performance in ice hockey players with those of strength training alone, in an 8-week study where 18 participants were randomly assigned to two groups.

Furthermore, functional testing is not routinely included in the periodic evaluation of young ice hockey players within club settings. The aim of this study was to investigate the effects of a FST program on sport-specific on-ice speed and agility in young elite male ice hockey players. By addressing movement dysfunctions, such as asymmetries and muscle imbalances, FST may reduce the physical demands of match play, mitigate the risk of micro-injuries and subsequent injuries, and ultimately enhance on-ice performance. Therefore, we hypothesized that FST would lead to significant improvements in performance measures.

## 2 Methods

### 2.1 Experimental approach to the problem

A quasi-experimental design was used to investigate the effect of a 12-week FST program on ice speed and agility in young elite ice hockey players. From a group of 43 hockey players, 22 were randomly assigned to the FST group (FSTG), while the remaining 21 were allocated to the control group (CG). The study consisted of three phases. The first phase involved pre-testing. The second phase was the intervention, during which the FSTG participated in supervised 60-min FST sessions twice per week. In contrast, the CG engaged in one 60-min team game session (volleyball, basketball, or soccer) and one 60-min swimming session per week. The third phase involved post-testing. The intensity of training in both groups was not monitored.

### 2.2 Participants

The study involved forty-three male elite ice hockey players, aged between 15 and 18 years (mean age 16.2 ± 0.76). These participants trained at the Hockey Sports School of the Polish Ice Hockey Federation in Katowice, Poland.

The sample size for this study was calculated using G*Power software version 3.1.9.7 developed by Heinrich Heine University in Düsseldorf, Germany. The calculations for a repeated measures ANOVA with a within-between interaction assumed an effect size *f* of 0.3, an alpha level of 0.05, and a test power of 0.96. Based on these parameters, the total required sample size was determined to be forty.

To qualify for this study, participants were required to meet the following inclusion criteria: a minimum of 7 years of competitive ice hockey experience, no injuries in the past 6 months, no absences from competitions exceeding 30 days, and no surgeries in the past 12 months.

The participants had previously undergone off-ice training as part of their regular regimen before enrolling in the FST program. This earlier training included traditional strength exercises performed on machines and with free weights, along with plyometric drills.

All the hockey players resided in the same dormitory, consumed the same meals, and did not participate in any other forms of physical activity apart from those planned by the coaches. Participants were initially instructed to avoid any physical activity beyond what was included in the designated training program. Prior to each FST session, a brief interview was conducted to clarify the purpose of the specific training session and outline the tasks to be performed. The explanation emphasized the relevance of the session to health, injury prevention, physical preparation, and movement quality enhancement. This approach aimed to motivate the athletes to train effectively.

Players were informed that training groups would be reorganized after the 12-week intervention to allow all participants, including those in the CG, the opportunity to engage in FST. However, they were not told that the tests were directly related to the training program. This approach was employed to minimize any potential competition between the FSTG and CG, thereby reducing bias in the study outcomes. Since the players were accustomed to regular testing, this did not represent a novel situation for them. The players had no prior experience with FST and had never performed it before.

The study was approved by the Bioethics Committee of the Jerzy Kukuczka Academy of Physical Education in Katowice (Certificate of Approval No. KB 2/2017) and conformed to the standards set by the Declaration of Helsinki. All participants and their parents/legal guardians were informed about the nature and the aim of the study, and they provided written informed consent before participating in the study.

### 2.3 Methods and procedures

Participants underwent anthropometric measurements, including body height (BH) and body mass (BM). BM was assessed using electrical impedance via the Tanita-410 Body Composition Analyzer (Tokyo, Japan). BH was measured with an anthropometer Tanita HR-001 (Tokyo, Japan). A professional Smart Speed measurement system (Fusion Sport, Coopers Plains, QLD, Australia), used by teams and training centers worldwide, was used to conduct specific fitness tests on the ice. The system comprises gates (each equipped with a photocell with an infrared transmitter and a light reflector) and a reader to identify the athlete. The participant starts from a standing position at any time, and the time is counted from the moment they pass the first photocell. Both forward and backward tests were performed twice, and the better result was used in the analysis. The two inter-times and the final total time were recorded. The inter-times illustrate how acceleration occurs in these athletes over the first 5 m (Grabara and Bieniec, 2024; Wagner et al., 2021).

Four tests from the IIHF (International Ice Hockey Federation) test database were utilized to assess selected aspects of specific on-ice fitness (Hockey Canada, 2014).

#### 2.3.1 5-, 15- and 30-m straight-line speed tests on ice forward and backward

The tests were conducted using four gates equipped with photocells. A 5-m distance was maintained between each photocell and its corresponding light reflector. The timing gates were positioned at the starting line, at 5 m and at 15 m to record intermediate times, and at 30 m to mark the finish line. Each player performed the test twice, and the better result was recorded for analysis. The time recorded at 5 m represented the athlete’s acceleration time and initial force output, while 15-m–marking the halfway point of the measured distance–indicated whether the athlete has reached peak acceleration speed, or if he was beginning to maintain or lose speed. Acceleration tends to decreases when the speed drops below 3 m/s at distance longer than 15 m (Grabara and Bieniec, 2024; Stastny et al., 2023). The total time at 30 m was also included in the analysis. Forward skating speed typically peaks between 25 and 39 m, with 30 m being the most commonly used and recommended distance. It is considered the most practical standard for game situations, as it reflects the ability to rapidly reach near-maximum speed (Stastny et al., 2023).

#### 2.3.2 Agility test on ice were conducted as follows

**FIGURE 1:**
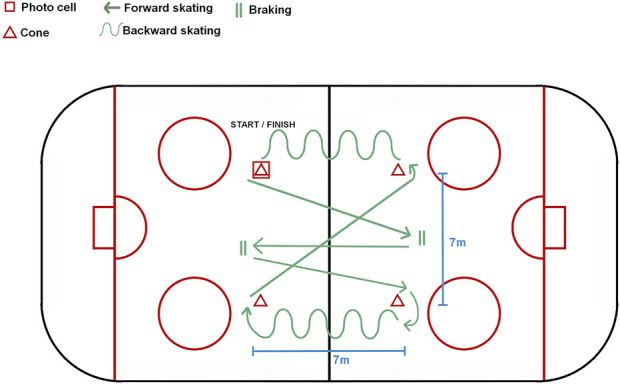
Agility skating transition.

The athlete starts from the starting line and skates to the far-right bollard, makes a right arc, and transitions to skating backwards. The athlete continues skating backwards to the near-right bollard, passes it, and transitions to forward skating. Then the athlete skates to the far-left bollard, makes a left arc, and transitions to skating backwards. Then, the player continues skating backwards to the near-left bollard, transitions to forward skating again, and skates to the line opposite the start/finish line. The player brakes at this line and then accelerates towards the finish line. This is a timed trial. Since the start line also served as the finish line, only one photocell was used for timing, positioned 2 m away from the light reflector (Figure 1) (Hockey Canada, 2014). Agility tests assess the efficiency of executing preplanned changes of direction, such as tight turns, braking, crossovers, and between skating techniques over short distances (Bournival et al., 2023). Therefore, after discussion with the coaches, we decided that, given the importance of these components for agility, the players would perform this test.

#### 2.3.3 Fastest lap test

**FIGURE 2:**
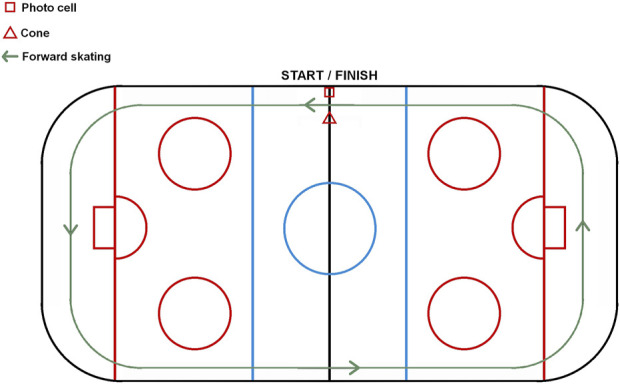
Fastest lap test.

The athlete completed a full lap of the rink, skating behind the net lines, as quickly as possible. Timing was conducted using a single photocell positioned at the start/finish line (Figure 2).

A rest period of approximately 10 min was provided between trials within each test, during which the entire group completed the respective test. After completing a test, the participants proceeded to rearrange the photocells and prepare for the subsequent test. During this preparation time, players skated slowly around the rink to maintain light activity.

### 2.4 Functional strength training program

The FSTG participated in a structured FST program over a 12-week period, consisting of two 60-min training sessions per week. These sessions were systematically planned and supervised by instructors to ensure precise execution of the exercises and adherence to program goals. Our prior study, which assessed movement patterns in this group of ice hockey players using the FMS test, identified notable limitations, primarily in mobility and stability (Bieniec and Grabara, 2024). These findings suggested that the athletes’ previous off-ice training may not have adequately addressed functional movement patterns. Consequently, a comprehensive FST program was implemented, beginning with the foundational components of the motor control pyramid: mobility, stability, and strength patterns. These components were progressively integrated with external loads and complemented by plyometric exercises.

The training program followed a systematic progression, starting with mobility and stability exercises and advancing to foundational movement patterns. Initially, these exercises were performed with body weight to emphasize proper technique and control, before gradually incorporating external resistance. The final stages of the program included plyometric exercises and track-and-field drills to enhance explosive power and athletic performance. Training sessions were conducted on Mondays and Thursdays under the joint supervision of a physiotherapist holding a National Certificate in Strength & Conditioning (NCSC) and technical coaches.

Specific training goals are outlined in the FST program:

#### 2.4.1 Mobility

An adequate level of mobility is essential for enhancing the quality of sensory stimulus reception, thereby facilitating the development of stabilization strategies and motor control (Cook et al., 2006a). A restricted range of motion (ROM) can impair movement quality (Bieniec and Grabara, 2024; Wilcox et al., 2015). Assessments performed on this group, including the FMS test, Y-Balance test, and physiotherapeutic evaluations such as the Dega, Patrick, and Thomas tests, revealed notable limitations in the shoulder and hip girdle complexes, as well as reduced dorsiflexion mobility in the ankle joints (Bieniec and Grabara, 2024; Grabara and Bieniec, 2023). Mobility exercises were executed slowly, ensuring full range of motion (ROM), with a controlled pause of 5–10 s at the end of the pain-free ROM. Various tools were utilized to support mobility training, including mats, rollers, duorollers, yoga straps, Swiss balls, sliding discs, power bands, and mini bands. This diverse range of equipment allowed for a comprehensive and targeted approach to addressing the identified mobility limitations.

#### 2.4.2 Stability

Stability is a fundamental characteristic for maintaining control of the body during both static and dynamic movements. Optimal proximal stability is essential for ensuring safe and effective distal movement (Cook et al., 2006b). The FST sessions were systematically designed to enhance postural stability, forming a solid foundation for the efficient functioning of the musculoskeletal system (Boyle, 2016; Gambetta, 2007). The exercises focused on uncompensated and controlled movement, providing a basis for introducing more complex patterns involving external resistance (Bieniec and Grabara, 2024; Boyle, 2016). Stabilization exercises were performed until the athlete lost balance or compensatory movements emerged. At that point, the coach provided corrections, allowing the athlete to return to the proper position and continue the exercise. For these exercises, a variety of tools were employed to enhance engagement and challenge stability, including BOSU balls, Swiss balls, TRX systems, sliding discs, medicine balls, and dumbbells.

#### 2.4.3 Integration of movement pattern

The integration of mobility and stability into specific movement patterns requires proper coordination and timing, including the sequential activation of muscles. This motor control encompassed mobility, stability, coordination, and effective muscle activation (Cook et al., 2006b).

#### 2.4.4 Strength and dynamic exercises

The program included bilateral and unilateral strength exercises, such as hip thrusts, single-leg hip thrusts, deadlifts, single-leg deadlifts, pull-ups, back and front squats, and landmine lateral squats, Cossack squats, lunges in all directions. Weights were individually tailored to each athlete to ensure exercises were performed through a full ROM without compensation. Initially, athletes trained using their body weight, with progression to external loads as tolerated. Athletes who were able to perform 6–8 repetitions with ease were encouraged to increase the weight, ensuring that each repetition maintained proper technique and full ROM. Time under tension (TUT) was emphasized, with athletes instructed to hold the end position of the movement for 3–5 s to enhance control and engagement. Training intensity was not directly measured; however, the load was adjusted so that the final repetition was challenging but achievable without compromising technique. Equipment used: bars, trapbars, kettlebells, dummbells, plates, weighted vests, medicine balls, ropes.

#### 2.4.5 Plyometric Training

Plyometric exercises were introduced after 4 weeks to enhance the efficiency of the stretch-shortening cycle (SSC), a critical mechanism for generating maximal power in minimal time, particularly relevant for acceleration. Proper jump technique was a primary focus, emphasizing full ROM, symmetrical movements, and stable landings to ensure safety and effectiveness. Plyometric drills were scheduled early in the week when athletes were well-rested and were performed at the start of training sessions, consisting of 15–25 jumps. The equipment used included coordination ladder, cups, hurdles (15 cm–1 m), and gymnastic sticks.

The training intensity was progressively increased across sessions. During the initial weeks, the focus was on mobility exercises, stability, and strength patterns without additional load, with an emphasis on extended TUT—up to 5 s—in the isometric phase at the end of movements. Athletes were continuously monitored and guided by the coaching staff to ensure proper execution. Coaches provided correction for any errors and encouraged athletes to fully engage in each repetition. When athletes demonstrated readiness, they transitioned to a group that incorporated additional loads while still maintaining the requirement for full movement execution, controlled tempo, and a pause at the end of each motion. Plyometric sessions were strategically scheduled after a day off or following a low-intensity on-ice training session to optimize recovery and performance. Initially, athletes were taught proper techniques for foundational plyometric movements such as squat jumps, countermovement jumps, and broad jumps. Once proficiency in these exercises was achieved, they progressed to more advanced drills like single-leg jumps. Plyometric sessions were often combined with coordination ladder exercises, wall drills, and running-strength drills. To prioritize quality over quantity, athletes were given longer rest periods between sets, allowing for maximum effort in each repetition. The shortened FT program is presented in Table 1.

**TABLE 1:** Shortened functional training program (FT).

Type of exercises	Workout methods	Training load
1. Mobilizations (applied from 1st week of FT)	Myofascial release using a foam roller and lacrosse ball; post-isometric relaxation (PIR) targeting muscles with increased tension (e.g., back muscles, shoulders, hip flexors, adductors, hamstrings); joints mobilizations for the shoulder and thoracic spine to improve chest opening, as well as for the hip flexors and rotators, and ankle dorsiflexion	4–6 exercises performed consecutively, 1 set of 6–8 repetitions for each exercise, with no breaks between exercises
2. Stabilization exercises (applied from 1st week of FT)	Static and dynamic balance exercises; activation of the deep muscles of the pelvic-hip-lumbar complex, gluteal muscles, and the rotator cone. Single-leg deadlifts, single-leg overhead presses, Swiss ball exercises for core stability, dead bug, bear dog, single-leg exercises with medicine ball, and yoga balance poses	4–6 exercises performed consecutively, 1 set of 6–8 repetitions for each exercise, with no breaks between exercises
3. Integration of movements patterns, exercises with extra load (applied from 4th week of FT)	Unilateral and bilateral squats, deadlift (DL), single-leg DL, hip thrusts (HT), single-leg HT, lunges, lateral squat, Cossack squat, pulling and pushing patterns, as well as rotational, and anti-rotational patterns (e.g., chopping and lifting)	3–5 exercises for unit, performed in 3–4 sets of 6–8 repetitions each exercise. Progressive loading applied, individual for every player, with 2 min break between sets. RPE 10
5. Agility exercises and plyometric exercises (applied from the 6th week of FT)	Track-and-field exercises, coordination ladder exercises, skips, half skips, wall drills, plyometric exercises (e.g., broad jumps, vertical jumps, hurdle jumps, unilateral jumps), and change-of-direction drills (COD)	4–6 exercises, performed in 4–6 sets, of 4–8 repetitions each exercise. RPE 7

### 2.5 Statistical analysis

Results are expressed as means, standard deviations (M ±sd), and differences between the pre- and post-test and between groups (Δ). The normality of the data distributions was assessed using the Kolmogorov-Smirnov test. Speed test results were analyzed with two factors: group (experimental or control) and time (pre- and post-test) using a two-way repeated measures ANOVA. When significant interactions were identified, the Bonferroni *post hoc* test was applied. Differences in body height and mass between experimental and control groups were analyzed using the t-test.

The significance level for all tests was set at α = 0.05. Empirical data were analyzed using Microsoft Excel and STATISTICA (version 13.3, TIBCO Software Inc. Palo Alto, CA, United States).

## 3 Results

The mean body height of the participants was 182.4 ± 6.06 cm, and the mean body mass was 80 ± 6.09 kg, with no significant differences between the groups.

The analysis of speed test results revealed varying effects across distances and predictors (Table 2).

**TABLE 2:** Results of ice speed in the experimental (E) and control (C) groups.

Speed test	Group	Mean ± sd			Predictors		
		Pre-test	Post-test	Δ	Time	Group	Time x group
5 m F	E	1.46 ± 0.37	1.32 ± 0.35	0.14 ± 0.47	*F* = 0.06 p = 0.81 *η*_*p*_^2^ = 0.001	*F* = 3.38 p = 0.076 *η*_*p*_^2^ = 0.076	***F* = 4.47** **p = 0.041** ***η***_***p***_^**2**^ **= 0.098**
	C	1.51 ± 0.39	1.62 ± 0.37	−0.11 ± 0.23			
15 m F	E	2.82 ± 0.37	2.61 ± 0.29	0.21 ± 0.30	***F* = 5.46** **p = 0.024** ***η***_***p***_^**2**^ **= 0.118**	*F* = 1.47 p = 0.232 *η*_*p*_^2^ = 0.035	***F* = 4.18** **p = 0.047** ***η***_***p***_^**2**^ **= 0.093**
	C	2.85 ± 0.5	2.84 ± 0.37	0.01 ± 0.32			
30 m F	E	4.27 ± 0.15	4.06 ± 0.31	0.21 ± 0.30	*F* = 2.73 p = 0.106 *η*_*p*_^2^ = 0.062	***F* = 11.63** **p = 0.001** ***η***_***p***_^**2**^ **= 0.221**	***F* = 7.19** **p = 0.011** ***η***_***p***_^**2**^ **= 0.149**
	C	4.37 ± 0.33	4.42 ± 0.25	−0.05 ± 0.34			
5 m B	E	1.95 ± 0.53	1.98 ± 0.58	−0.03 ± 0.66	*F* = 1.56 p = 0.219 *η*_*p*_^2^ = 0.037	*F* = 0.01 p = 0.907 *η*_*p*_^2^<0.001	*F* = 0.88 p = 0.353 *η*_*p*_^2^ = 0.021
	C	1.85 ± 0.59	2.04 ± 0.48	−0.2 ± 0.49			
15 m B	E	3.24 ± 0.43	3.09 ± 0.64	0.15 ± 0.66	*F* = 1.72 p = 0.197 *η*_*p*_^2^ = 0.04	*F* = 2.66 p = 0.11 *η*_*p*_^2^ = 0.061	*F* < 0.01 p = 0.938 *η*_*p*_^2^<0.001
	C	3.47 ± 0.73	3.34 ± 0.56	0.13 ± 0.72			
30 m B	E	5.18 ± 0.26	5.10 ± 0.39	0.08 ± 0.28	*F* < 0.01 p = 0.957 *η*_*p*_^2^<0.001	*F* = 3.52 p = 0.068 *η*_*p*_^2^ = 0.079	*F* = 1.41 p = 0.241 *η*_*p*_^2^ = 0.033
	C	5.32 ± 0.42	5.39 ± 0.6	−0.07 ± 0.53			
Agility	E	14.39 ± 0.61	14.14 ± 0.74	0.25 ± 0.49	***F* = 5.58** **p = 0.023** ***η***_***p***_^**2**^ **= 0.12**	***F* = 11.98** **p = 0.001** ***η***_***p***_^**2**^ **= 0.226**	*F* = 1.93 p = 0.173 *η*_*p*_^2^ = 0.045
	C	15.16 ± 1.04	15.09 ± 0.92	0.07 ± 0.39			
Full Speed	E	15 ± 0.75	15.03 ± 0.74	−0.03 ± 0.40	*F* = 1.83 p = 0.183 *η*_*p*_^2^ = 0.043	***F* = 5.37** **p = 0.026** ***η***_***p***_^**2**^ **= 0.116**	*F* = 0.91 p = 0.346 *η*_*p*_^2^ = 0.022
	C	15.46 ± 0.84	15.61 ± 0.76	−0.16 ± 0.48			

Notes: F–forward; B-backward; M ±sd–mean and standard deviations; Δ - pre-to-posttest difference; *F*–*F(1, 41)*; ANOVA, statistic with 1 degree of freedom for the effect and 41 for the error term; *η*_*p*_^2^ – the partial eta squared.

Significant values (p < 0.05) are in bold.

For the 5-m forward skating test, a significant interaction between time and group was detected, suggesting differential changes between groups over time, with a moderate effect size. However, the main effects of time and group were not significant, indicating minimal independent contributions from these predictors. Further analysis using Bonferroni-adjusted *post hoc* tests did not identify specific significant differences between time point or group. In the 15-m forward test, a significant interaction effect and a significant main effect of time were observed, reflecting moderate effect sizes and suggesting differing responses between group. Post hoc analysis revealed a significant difference in the experimental group between pre- and post-test (Δ = 0.21 ± 0.7, CI [0.02–0.39], *p* = 0.019) indicating that the hockey players assigned to the experimental group improved their ice speed and achieved significantly better results in the15-m forward test. A similar trend was observed in the 30-m forward test, where a significant main effect of group and a significant interaction between time and group were detected, both reflecting large effect sizes. Post hoc analysis revealed a significant improvement between pre and post-test in the experimental group [Δ = 0.21 ± 0.07, CI (0.02–0.4), *p* = 0.021], and a significant difference between the experimental and control groups in the post-test [Δ = 0.36 ± 0.08, CI (0.13–0.53), *p* < 0.001].

Conversely, for the 5-m backward, 15-m backward, and 30-m backward tests, no significant main effects or interaction effects were observed.

For agility performance, significant main effects of time and group were found, indicating moderate and large effect sizes, respectively, while the interaction effect was non-significant. Post hoc analysis revealed statistically significant differences between the experimental and control groups at both the pre-test [D = 0.76 ± 0.26, CI (0.05–1.47), *p* = 0.028] and post-test [D = 0.95 ± 0.26, CI (0.24–1.66), *p* = 0.003].

Finally, in the full speed test, a significant main effect of group was identified, indicating a moderate effect size. Neither the time effect nor the interaction effect was significant. Post hoc tests did not reveal specific significant differences between time points or groups.

## 4 Discussion

The study investigated the effects of the FST program on ice speed and agility in young male elite ice hockey players. The hypothesis that the FST would enhance performance measures was partially supported by the results. Over time, several improvements were observed in the FSTG. Specifically, significant decreases were noted in the time required to skate 15- and 30-m forward following the implementation of FST. A significant interaction effect between time and groups was observed in all forward skating results, emphasizing the positive influence of the intervention on performance changes in the FSTG compared to the CG. The targeted exercises in the FST program may have contributed to these improvements in forward skating speed. However, no significant enhancement was observed in backward skating performance in either the FSTG or CG. This lack of improvement could potentially be attributed to the low frequency of high-intensity backward skating exercises during on-ice training sessions (Ebben et al., 2004; Roczniok et al., 2024). As demonstrated in the study by Brocherie et al. (Brocherie et al., 2018) during a game, slow backward skating accounted for approximately 9% of the total effective time, whereas fast backward skating and sprinting constituted only 3% of the total effective time. In contrast, forward skating occupied a considerably larger proportion, with 33% of the total effective time spent on slow forward skating, 12% on fast forward skating, and 5% on forward sprinting (Brocherie et al., 2018). The initiation of backward movement requires effective activation of deep stabilizing muscles, primarily the abdominal muscles, as well as the hamstring muscles, alongside the efficient functioning of the ankle joint.

The study involving the same ice hockey players examined the effects of a 12-week functional training program on basic movement patterns and dynamic balance, demonstrating significant improvements in both areas (Bieniec and Grabara, 2024). After completing the functional training program, the hockey players showed better results in the deep squat, hurdle step, in-line lunge, shoulder mobility, and rotary stability tests, had higher total FMS scores, and exhibited fewer asymmetries compared to baseline. Conversely, the hockey players who did not participate in the functional training program showed a decrease in performance in the hurdle step and an increase in asymmetries compared to baseline (Bieniec and Grabara, 2024). The enhancement of fundamental movement patterns may have translated into improved skating performance, as indicated by the results of the current study. Previous studies have shown a correlation between speed on ice and selected off-ice motor tests (Bracko, 2009; Dæhlin et al., 2017). Sprints, vertical and horizontal jumps with both feet and one leg, some strength patterns, such as squats, hang power clean with a maximum load of 1-3 repetitions performed with a maximum load (RM) showed a positive correlation with speed on ice and ice hockey performance potential (Bracko, 2009; Dæhlin et al., 2017; Laakso and Schuster, 2021; Novák et al., 2019).

Strength training and weightlifting are commonly used as a training method for hockey players to develop strength, speed and explosiveness (Laakso and Schuster, 2021). Performing these exercises correctly can contribute to the development of lower limb power required for fast skating (Edman and Esping, 2013). If athletes have limitations in movement and motor control, compensations can occur and the athlete may not perform the exercise through its full range of motion (ROM). This can lead to overloading the overused body parts even more compensatively and not fully engaging the intended muscle groups. In training programming, many authors emphasize that the first step in functional training is to achieve adequate ROM in the joints; the next step is to ensure control of that movement, especially in its outer ranges (Boyle, 2016). Therefore, improving the squat pattern seems reasonable to enhance specific skills on the ice. Previous studies have found that increases in squat strength correlate with both off-ice sprinting (Bouteraa et al., 2020) and on-ice sprinting (Seitz et al., 2014). This relationship may be due to force generation around the same joint angles, as both squatting and sprinting on ice and on the field require simultaneous extension of the hip, knee and ankle joints (De Koning et al., 1995). Squatting with the thighs parallel to the ground results in hip flexion angles of approximately 40–45^°^, similar to those observed in forward speed skating (Bracko, 2009). Functional limitations in range of motion often prevent hockey players from achieving this depth, making functional training a good method to improve strength patterns and activate the appropriate muscle groups within these patterns.

As an extensor of the knee, the quadriceps muscle plays an important role in the movement of speed skating (Upjohn et al., 2008). A study by Robertson et al. (Robertson et al., 2008) also showed increased EMG activity of the quadriceps muscle of the thigh in a deep squat when the thighs are parallel to the ground, i.e., when the knee joint flexion is about 90°. Peak quadriceps activity occurs at 90 degrees of knee joint flexion. Optimal squat depth also results in activation of the gluteus maximus muscle and the ankle flexors whose contribution to the acceleration phase of skating is significant (Upjohn et al., 2008).

Previous studies have suggested that limited ankle dorsiflexion mobility results in reduced peak knee flexion, which may generate compensatory movements for proper squat execution and prevent full activation of the knee extensors (Kritz et al., 2009). Improving ankle dorsiflexion mobility can enhance squat depth. An athlete who can squat deeper while maintaining control of the movement will be able to better engage the lower limb muscles during this exercise. By strengthening the quadriceps, gluteus maximus, and ankle flexors, athletes can demonstrate greater power and speed on the ice (Kasuyama et al., 2009).

The optimal ROM in the lower limb joints facilitates a lower center of gravity and a more stable body position (Spiteri et al., 2013). Enhanced mobility in the hip and knee joints can improve the stretch-contraction cycle of the gluteus maximus muscle, thereby stimulating the quadriceps to contract concentrically during the push-off phase. This can lead to the generation of greater power and consequently, increased acceleration (Buckeridge et al., 2015; De Koning et al., 1995). Similarly, optimal ankle joint mobility can contribute to greater power generation during the push-off and glide phases. Optimal dorsiflexion range of the ankle joint during skate glide allows for the triceps calf muscle to stretch, which can translate through the stretch-contraction cycle into better power generation during the push-off phase of soleus flexion (Robbins et al., 2021). Faster skaters primarily pushed off with the forefoot, while weaker skaters distributed forces between the forefoot and midfoot. Greater hip extension and adduction enabled skaters to better utilize the forefoot and push off more effectively (Buckeridge et al., 2015). It can be inferred that faster hockey players exhibited a greater ROM of their lower extremities, leading to improved skating patterns (Robbins et al., 2021). According to Novák (Novák et al., 2019), faster hockey players demonstrated greater hip and knee ROM during the push-off phase. These ROMs may influence the lengthening of the skating stride and increase skating speed (Buckeridge et al., 2015; Upjohn et al., 2008). Faster hockey players were also characterized by quicker hip inversion, knee extension and ankle plantar flexion during the push-off phase (Robbins et al., 2021). Athletes at higher league levels have been shown to possess greater ROM in hip flexion, extension, rotational movements, knee flexion, and ankle dorsiflexion compared to hockey players in lower leagues (Buckeridge et al., 2015; Robbins et al., 2021).

Due to the flexed body position, hockey players may exhibit tight hip flexor muscles and weak extensor muscles. Consequently, tight hip flexors may inhibit or reduce the activation of the gluteal muscles, which play a pivotal role in the acceleration phase (Page, 2002). The hip flexor relaxation and mobilization exercises performed in the experimental group classes may have contributed to an improved ROM in hip extension and activated the gluteal muscles.

Following the 12-week FST program, significant main effects were observed for both time and group, indicating overall improvements in agility performance over time and differences between the FSTG and CG, respectively. However, the lack of a significant interaction effect suggests that the observed improvements in agility were not attributable to differential rates of change between groups over time. This indicates that the FST program had a beneficial impact on agility performance, although the pattern of improvement over time was consistent across both groups. Wu et al. (2023) also observed that 8 weeks of functional training can improve speed, and agility. Agility plays a key role in ice hockey training. It is defined as the ability to change direction quickly depending on the game situation. Often, with a high speed of movement and in contact with the opponent, agility can provide a physical and tactical advantage over the opponent (Novák et al., 2019). Most movements require athletes to exert alternating force on one leg while running, skating or changing direction (Gonzalo-Skok et al., 2017). When a hockey player has better control over movement, he has a greater ability to generate power and skating speed. It has been shown that the dynamic balance of young hockey players has a significant relationship with maximum skating speed (Behm et al., 2005). It was observed that the addition of balance training elements improved jump height and landing control and also had a positive effect on agility (Salaj et al., 2011).

Strength and endurance training, commonly utilized in ice hockey, may not be sufficient for comprehensive athletic development. According to Slavicek et al. (Slavicek et al., 2022) players with greater lower limb skeletal robustness relied primarily on strength and endurance to enhance their change of directional speed (COD) performance. In contrast, players with lower skeletal robustness exhibited greater reliance on technique and skill, demonstrating superior puck control, which necessitates well-rounded motor abilities (Slavicek et al., 2022).

Improved balance is correlated with greater jump distances, which, as indicated by a previous study, are strongly associated with forward skating speed (Lockie et al., 2015). Sprint skating has been found to be strongly correlated with plyometric testing (Gupta et al., 2023). The three-distance jump test and 30-m off-ice sprint are correlated with on-ice speed (Dæhlin et al., 2017). The introduction of plyometric exercises was gradual, commencing with basic bilateral vertical jumps. Subsequently, hurdles were utilized to enhance height, followed by long jumps, and ultimately, single-leg jumps. The implementation of plyometric training aimed at augmenting ground reaction force, as measured by the Reactive Strength Index (RSI), can potentially enhance acceleration (Dæhlin et al., 2017). Adaptation to plyometric training may increase tendon stiffness, enhancing force development by enabling more efficient force transfer to the bones. This can boost ground force during brief ice contact times in skating acceleration, where forefoot muscle strength is vital. Consequently, stiffening the ankle soleus flexors’ musculotendinous elements can enhance lower limb strength and power, leading to increased speed (Buckeridge et al., 2015; Dæhlin et al., 2017).

It is also important to consider that the FSTG players were not accustomed to this type of functional training with load progression aimed at reinforcing the correct movement pattern. Such training may have acted as a strong, sudden stimulus, leading to improvements in sport-specific performance on the ice. In contrast, elite athletes of the same age who regularly participate in conditioning camps with systematically planned motor training may not exhibit such performance differences (Ebben et al., 2004), as indicated in our study. Roczniok et al. (Roczniok et al., 2024) demonstrated that sub-elite athletes exhibited a stronger relationship between on-ice and off-ice test results compared to elite athletes, whose skating sprint was less dependent on their ability to generate propulsive force in both vertical and horizontal directions. This effect can be attributed to the higher level of skating technique among elite players, as reflected by a lower skating efficiency index (Roczniok et al., 2024). The potential influence of differences in skating technique is further supported by previous studies, which have shown that elite athletes benefit from a greater functional range of motion and superior horizontal power transfer (Budarick et al., 2020; Slavicek et al., 2022).

### 4.1 Strengths and limitations of this study

This study provides valuable insights into sports training, specifically examining the impact of the FST program on enhancing speed and agility in young ice hockey players. However, there are several limitations to this study. The first limitation is the inability to conduct a follow-up study after a period of 6 weeks. The second limitation is the lack of monitoring the intensity of both FST and other exercises undertaken by the participants, which could introduce bias. In future studies, it would be essential to consider this aspect. Additionally, the inability to monitor participants’ sleep patterns represents another limitation. Another limitation is the narrow age range of the athletes studied, restricted to 15–18 years. Therefore, caution is warranted when generalizing these findings to a broader population of athletes.

## 5 Conclusion

The findings of this study demonstrate that a 12-week FST program significantly improved ice speed and agility in young hockey players in the FSTG, whereas no comparable improvements were observed in the CG. These results underscore the effectiveness of FST in enhancing forward speed and overall athletic performance in young ice hockey players. These findings suggest and support coaches in youth teams should consider similar training programs to adapt athletes to the demands of elite-level competition.

## Data Availability

The raw data supporting the conclusions of this article will be made available by the authors, without undue reservation.
